# The association between socioeconomic distress communities index and amputation among patients with peripheral artery disease

**DOI:** 10.3389/fcvm.2022.1021692

**Published:** 2022-11-03

**Authors:** Brian Witrick, Lu Shi, Rachel Mayo, Brian Hendricks, Corey A. Kalbaugh

**Affiliations:** ^1^West Virginia Clinical and Translational Science Institute, Morgantown, WV, United States; ^2^Department of Public Health Sciences, Clemson University, Clemson, SC, United States; ^3^Department of Epidemiology and Biostatistics, West Virginia University School of Public Health, Morgantown, WV, United States; ^4^Department of Epidemiology and Biostatistics, Indiana University School of Public Health, Bloomington, IN, United States

**Keywords:** peripheral artery disease (PAD), amputation, distressed communities index (DCI), socioeconomic, health disparity

## Abstract

**Background:**

Socioeconomic factors have been shown to be associated with amputation in peripheral artery disease (PAD); however, analyses have normally focused on insurance status, race, or median income. We sought to determine whether community-level socioeconomic distress was associated with major amputation and if that association differed by race.

**Materials and methods:**

Community-level socioeconomic distress was measured using the distressed communities index (DCI). The DCI is a zip code level compositive socioeconomic score (0–100) that accounts for unemployment, education level, poverty rate, median income, business growth, and housing vacancies. A distressed community was defined as a zip code with DCI of 40 or greater. We calculated one-year risk of major amputation by DCI score for individuals with peripheral artery disease in South Carolina, 2012–2017. Treating death as competing event, we reported Fine and Gray subdistribution hazards ratios (sdHR), adjusted for patient demographic and clinical comorbidities associated with amputation. Further analyses were completed to identify potential differences in outcomes within strata of race and DCI.

**Results:**

Among 82,848 individuals with peripheral artery disease, the one-year incidence of amputation was 3.5% (95% CI: 3.3%, 3.6%) and was significantly greater in distressed communities than non-distressed communities (3.9%; 95% CI: 3.8%, 4.1% vs. 2.4%; 95% CI: 2.2%, 2.6%). After controlling for death and adjusting for covariates, we found an increased hazard of amputation among individuals in a distressed community (sdHR: 1.25; 95% CI: 1.14, 1.37), which persisted across racial strata. However, regardless of DCI score, Black individuals had the highest incidence of amputation.

**Conclusion:**

Socioeconomic status is independently predictive of limb amputation after controlling for demographic characteristics and clinical comorbidities. Race continues to be an important risk factor, with Black individuals having higher incidence of amputation, even in non-distressed communities, than White individuals had in distressed communities.

## Introduction

Peripheral artery disease (PAD) is a progressive circulatory disorder that occurs when arteries carrying blood from the heart to the legs are obstructed (1). PAD currently affects between 8 million to 12 million adults in the United States, but that number is expected to increase as the population ages and the prevalence of PAD risk factors increases (2–4). Individuals with PAD have reduced quality of life (5, 6) and increased risk for functional limitation (7, 8), cardiovascular disease (9, 10), and mortality (11, 12). Major limb amputation is a serious complication of PAD that arises when significant tissue loss has occurred, when medical or surgical intervention is not possible, or when an intervention has been unsuccessful (13, 14). Some amputations are medically necessary, usually in individuals with PAD who have chronic limb threatening ischemia. However, amputation is generally considered a failure of treatment and should only be attempted after considering all other treatment options. The majority of amputations are preventable, especially among individuals with PAD who have claudication, with timely diagnosis and optimal medical treatment (15, 16).

Socioeconomic and demographic characteristics are important factors that contribute to an individual’s access to medical care and health outcomes (17). Amongst individuals with PAD, low socioeconomic status is associated with poor access to care (18, 19), increased risk of hospitalization (20), and high prevalence of comorbid conditions (20, 21). Similarly, people from racial minority groups also have higher incidence of PAD (22), are disproportionately affected by poor access to care (1, 19), and have worse outcomes (23, 24). As more granular measures of economic status are often unavailable, race is commonly used as a proxy for SES (2, 25). We argue that race and socioeconomic status (SES) have a distinct process through which they impact health and there may be important differences that are ignored when SES and race are conflated (26).

To date, most socioeconomic disparities in amputation risk are based on individual-level (27) measures of SES or a specific socioeconomic component, such as median household income (28, 29). Comprehensive SES measures may improve predictions but are rarely included in models (30). In this study we utilize a composite ranking that incorporates social, economic, and financial metrics at the zip code level. The objectives of this study were to (1) investigate the association between community-level socioeconomic distress and major amputation among individuals with peripheral artery disease; and (2) determine if the association differed by race.

## Materials and methods

### Study population and data sources

The present study used data from the South Carolina Patient Encounter database (SCPED; 2010–2018) obtained from the South Carolina Revenue and Fiscal Affairs Office. Detailed information about the SCPED and the data collection process has been described previously (31). Briefly, the SCPED serves as the central repository of all health and human services data in South Carolina, including claims and administrative data from all hospital inpatient and outpatient facilities within the state. Every individual is assigned a unique identifier that is used to link across multiple providers and data sources and allows for unduplicated cases across the data systems care. This study was approved by the Clemson University Institutional Review Board (IRB2020-035).

Our study population included individuals aged 18 years or older who were admitted to an inpatient hospital or outpatient surgery facility in South Carolina for an index PAD encounter. PAD diagnosis was detected using ICD-9/10 codes (Supplementary material) and included individuals with claudication and chronic limb threatening ischemia A two-year lookback period was chosen to minimize misclassification of PAD diagnosis (32, 33). Individuals were followed for one year after index encounter. Patients who resided outside the state, had missing zip code, or for which socioeconomic distress could not be measured were excluded (7.3%; *n* = 6,559).

### Study measurements

Socioeconomic distress was measured using the distressed communities index (DCI). Developed by the Economic Innovation Group, the DCI incorporates seven metrics to estimate socioeconomic distress of a community at the zip code level (34). A DCI score is available for every zip code with at least 500 individuals, which captures 99% of the US population. The metrics include no high school degree, housing vacancy rate, adults not working, poverty rate, mean income ratio, change in employment, and change in business establishments (Supplementary material). Each variable is equally weighted, compared to its nearest neighbors, and normalized to obtain a raw score (35). A score of 0 indicates that a community has no distress and a score of 100 indicates severe distress. Based on the distribution of the population by DCI score, a distressed community in this study was defined as a zip code with DCI score **≥**40.

The primary outcome for this study was major limb amputation after index PAD encounter. Major limb amputation was defined as above or below the knee amputation, with toe and forefoot amputations excluded, and identified using ICD-9/10 procedural codes (Supplementary material).

Relevant covariates were identified *a priori* based on prior knowledge, published literature, and data availability. Demographics include age at index encounter, gender, and race (black/white). Payor was categorized as Medicare, Medicaid, Self-Pay, Private, and Other (HMO, Charitable/Indigent Organization, Worker’s Compensation). Clinical variables were identified during the two-year look period and at the index encounter using ICD 9/10 codes (Supplementary material). These variables included diabetes, renal failure, chronic obstructive pulmonary disease (COPD), congestive heart failure (CHF), coronary artery disease (CAD), chronic limb threatening ischemia (CLTI), and the Charlson comorbidity index (CCI).

### Statistical analysis

We compared the distribution of demographic characteristics, comorbid conditions, and study outcomes across DCI groups. Normally distributed continuous variables were reported as mean ± standard deviation and statistical differences were identified using independent *t*-test. Non-normally distributed continuous variables were reported as median and interquartile range and statistical differences were identified using the Mann-Whitney test. Categorical variables were presented as proportions and the chi-square test was performed to identify differences across DCI groups.

Individuals were followed from index PAD encounter until the first occurrence of major amputation or death (a competing event, which precludes the occurrence of major amputation). Data was administratively censored at one year. We presented crude cumulative incidence curves of amputation free survival, stratified by DCI group, to describe the differences in amputation outcomes after index PAD encounter (36). Differences between DCI groups were assessed using Gray’s test (37).

We used Fine and Gray subdistribution proportional hazards models to calculate subdistribution hazard ratios (sdHR) for the association between DCI score and major amputation, accounting for death as a competing event and adjusting for the independent variables listed above (38). An interaction term for race and DCI score was added to the model, but found to be non-significant; therefore, we report only the results of the model without the interaction term. In sensitivity analysis, the DCI score was scaled by 25 so that every 1-point change represents a 25-point (quartile) increase in the DCI (28).

All analyses were performed using SAS version 9.4 software (SAS Institute, Cary, NC), with a *P* < 0.05 set as statistically significant.

## Results

We identified 82,848 individuals who had an index PAD encounter at an inpatient hospital or outpatient surgery facility in South Carolina from 2012 to 2017. There were 57,196 (69%) individuals who resided in distressed communities and 25,652 (31%) who resided in non-distressed communities. The majority of the cohort were white (70%) and male (53%) with a mean age of 67 ± 14.1 years (Table 1). Individuals from non-distressed communities were more likely to be older, white, and have Medicare or Private insurance. Individuals from distressed communities were more likely to have diagnosed comorbidities, including diabetes, rental failure, COPD, and CHF.

**TABLE 1:** Demographics and clinical characteristics by distressed communities index (DCI) score.

	All patients	DCI < 40	DCI ≥ 40	*P*-value
	(82,848)	(25,652)	(57,196)	
Age, mean (SD)	67.0 (14.1)	68.3 (13.9)	66.4 (14.2)	<0.001
Female	38,982(47.1)	11,614(45.3)	27,368(47.9)	<0.001
Race/Ethnicity				<0.001
Non-hispanic white	57,744(69.7)	21,308(83.1)	36,436(63.7)	
Non-hispanic black	25,104(30.3)	4,344(16.9)	20,760(36.3)	
Primary insurer				<0.001
Medicare	27,247(69.1)	17,969(70.1)	39,278(69.7)	
Medicaid	5,089(6.1)	1,018(4.0)	4,071(7.1)	
No insurance	3,855(4.7)	977 (3.8)	2,878(5.0)	
Private	11,873(14.3)	4,057(15.8)	7,816(13.7)	
Other	4,784(5.8)	1,631(6.4)	3,153(5.5)	
Diabetes	45,496(54.9)	13,161(51.3)	32,335(56.5)	<0.001
Renal failure	9,742(11.8)	2,747(10.7)	6,995(12.2)	<0.001
COPD	20,234(24.4)	5,817(22.7)	14,414(25.2)	<0.001
CHF	18,039(21.8)	5,159(20.1)	12,880(22.5)	<0.001
CAD	31,776(38.4)	9,968(38.9)	21,808(38.1)	0.046
CLTI	17,237(20.8)	5,249(20.5)	11,988(21.0)	0.103
CCI, median (IDR)	2.0 (3.0)	2.0 (3.0)	2.0 (3.0)	<0.001

COPD, chronic obstructive pulmonary disease; CHF, congestive heart failure; CAD, coronary artery disease; CLTI, chronic limb threatening ischemia; CCI, Charlson comorbidity index.

**FIGURE 1:**
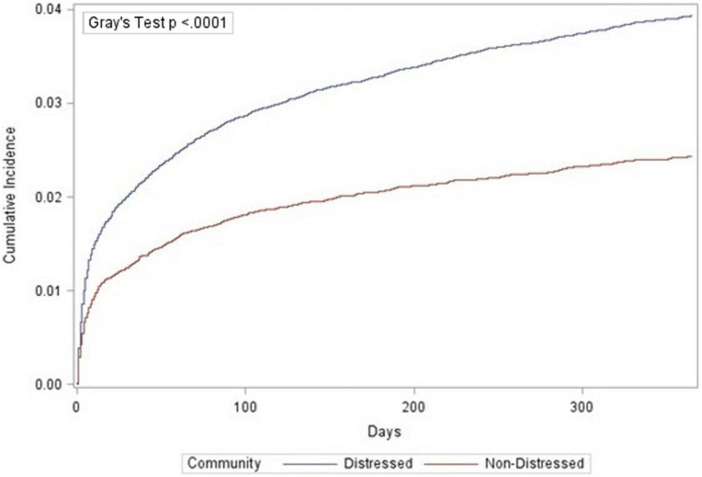
Cumulative incidence function of amputations by community distress level.

**FIGURE 2:**
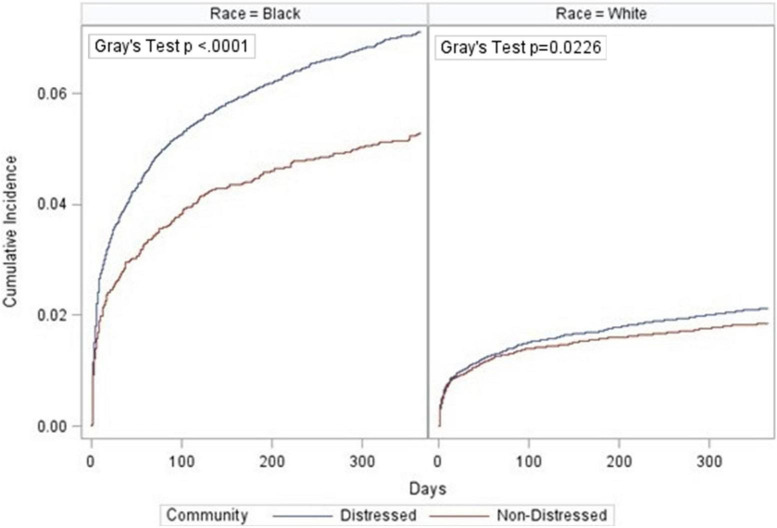
Cumulative incidence function of amputations by community distress level and race.

Among all PAD individuals, one-year cumulative incidence of major amputation was 3.5% (95% CI: 3.3%, 3.6%). The cumulative incidence of major amputation was significantly greater in individuals from distressed communities (3.9%; 95% CI: 3.8%, 4.1%) compared to non-distressed communities (2.4%; 95% CI: 2.2%, 2.6%; Figure 1). Among White individuals, incidence of major amputation ranged from 1.8% (95% CI: 1.7%, 2.0%) in non-distressed communities to 2.2% (95% CI: 2.0%, 2.3%) in distressed communities (Figure 2). Among Black individuals, incidence of major amputation was 5.3% (95% CI: 4.6%, 6.0%) in non-distressed communities and 7.1% (95% CI: 6.8%, 7.5%) in distressed communities.

**FIGURE 3:**
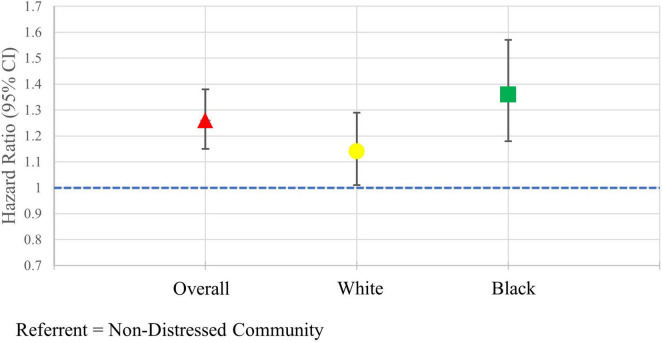
Adjusted hazard ratios of major amputation in the overall population and stratified by race.

After adjusting for demographic and clinical covariates, individuals who lived in distressed communities had a hazard of major amputation 1.25 (95% CI: 1.14, 1.37) times the hazard among individuals in a non-distressed community (Figure 3). Among White individuals, the sdHR comparing individuals in distressed communities to non-distressed communities was 1.14 (95% CI: 1.01, 1.29). Black individuals who lived in distress communities had an adjusted hazard of major amputation 1.36 (95% CI: 1.18, 1.57) times the hazard of Black individuals living in non-distressed communities. Similar results were seen in the sensitivity analysis when the DCI score was scaled by 25. The sdHR comparing individuals in distressed communities to non-distressed communities was 1.09 (95% CI: 1.05, 1.13) in the overall population, 1.07 (95% CI: 1.02, 1.13) in White individuals, and 1.09 (95% CI: 1.04, 1.14) in Black individuals.

## Discussion

In our study of more than 80,000 individuals with PAD, we found that DCI score is independently predictive of limb amputation after controlling for demographic characteristics and clinical comorbidities. The increased amputation risk was found across racial strata indicating the importance of an individual’s community for all races. The increased risk was greater in Black individuals than White individuals. Additionally, we also showed that Black individuals had higher incidence of amputation, even in non-distressed communities, than White individuals had in distressed communities. Race continues to be an important predictor of amputation risk among individuals with PAD, even after controlling for SES. Future studies should incorporate both race and SES to avoid missing important differences in outcomes when race is used as a proxy for SES.

Socioeconomic disparities among individuals with PAD have been well documented (39). Individuals with lower SES are disproportionately affected by PAD risk factors (40), have a higher risk of being hospitalized for PAD (20), and have worse outcomes (18). However, previous studies often focus more on individual level SES and may miss the important effects of where patients live. Our study contributes to a growing body of evidence that suggests that community-level socioeconomic characteristics also play a critical role in individuals with PAD (21, 28). Our findings confirm results from a recent study among veterans with PAD, extending these findings to highlight the importance of community-level socioeconomic disparities in amputation risks among different PAD populations (21). Furthermore, our study found that these community-level disparities persisted across racial groups, even after controlling for insurance status, which is an important determinant in access to health care. Our findings confirm that both individual-level and community-level SES are important factors in health disparities among individuals with PAD.

There are several possible mechanisms behind the association between community-level SES and amputation. Living in a distressed community exerts psychosocial stress on individuals due to high poverty, high unemployment, high crime, limited green space, and lack of access to nutritional food, amongst other contributing factors (39, 41, 42). Cumulative stress in these individuals correlates with the development and progression of atherosclerosis in the lower extremities, which is the main cause of PAD (43, 44). Furthermore, compared to non-distressed communities, distressed communities often have limited access to care and less availability of health care resources, such as vascular specialty care, that make it difficult to receive timely diagnosis and up-to-date management of PAD (28). Taken together, individuals that live in distressed communities are more likely to develop PAD due to cumulative stress, but less likely to have the necessary resources to manage it.

We identified a racial disparity in amputation risk among individuals with PAD, a finding that has been documented for decades (29, 45, 46). Racial disparities are believed to be due to differences in SES, insurance coverage, disease severity at presentation, and comorbid conditions (24). However, even after adjusting for these potential factors, we still observed that race was a major independent predictor of amputation. Further, we found that Black individuals in non-distressed communities had higher rates of amputation compared to White individuals in distressed communities. These findings indicate that regardless of the community they reside in, Black individuals are at higher risk of worse limb outcomes than Whites. Provider bias in clinical decision making and medical management of PAD has been explored in recent studies as a potential contributing factor in racial disparities (1, 21, 24). Although provider bias may partially explain the persistent differences in amputations rates among races, we are not able to properly explore this potential factor due to data limitations.

Structural racism has been identified as an important contributor to health disparities in the United States (47). Historically, racial minorities, particularly Black individuals, have been segregated to less-desirable neighborhoods that have higher concentrations of poverty. In these areas, Black individuals are exposed to more air pollution, have fewer job and educational opportunities, and have lower access to green space (48). As a result, it is more difficult for these individuals to practice healthy behaviors, such as adequate physical activity and proper nutrition, that can help prevent PAD. Living in these distressed neighborhoods also decreases their access to primary care, specialty care, and pharmacy services, which are necessary to manage chronic conditions that are comorbidities of PAD (16). Structural racism is particularly relevant in our study, which is based on data from South Carolina. We found that the proportion of Black individuals that reside in distressed communities is almost double that of non-distressed communities. Future analysis will explore the impact of structural racism through the inclusion of index of concentrations (ICE) at the zip code level (49).

Addressing the racial and socioeconomic disparities in amputation risk among individuals with PAD will need to involve coordinated efforts across multiple stakeholders (50). Our findings suggest that interventions need to be targeted on distressed communities, particularly those with a large Black population. Policy makers should invest in the development and implementation of community-based interventions that increase awareness of PAD and promote healthy behaviors. Community screening programs and exercise programs have proven to be effective in identifying individuals with PAD and improving functionality among these individuals (51, 52). Additionally, community and patient engagement interventions have proven to be effective in decreasing racial disparities in amputations among individuals with diabetes and may be adapted to the address such disparities in PAD (53).

There are several limitations in the study that should be noted. Our study is based on administrative claims data, which is subject to errors, inaccuracies, and discrepancies in coding practices (54). Residual confounding due to undocumented and undiagnosed relevant medical conditions can bias results. It is also possible that some of socioeconomic disparity found in this study is due to variations in hospital related practices and not the community level SES. Furthermore, this study assessed community-level SES using DCI scores at the zip code level. The DCI scores are only calculated for zip codes with > 500 people and were not available for every individual. Finally, although the DCI score is an accurate assessment of community-level SES, not every individual within a given zip code will be impacted the same way. Individuals with a high individual-level SES may be able to overcome the impacts of lower community-level SES.

## Conclusion

Individuals who reside in distressed communities had increased risk for amputation, even after adjusting for race and clinical conditions. Race continues to be an important predictor of amputation, with Black individuals having higher rates of amputation than White individuals, regardless of the SES of their communities.

## Data availability statement

The data analyzed in this study is subject to the following licenses/restrictions: The datasets analyzed for this study were obtained from the South Carolina Revenue and Fiscal Affairs Office and are available from the authors with the permission of the South Carolina Revenue and Fiscal Affairs Office. Requests to access these datasets should be directed to https://rfa.sc.gov/boards-committees/dataoversight.

## Ethics statement

The studies involving human participants were reviewed and approved by Clemson University Institutional Review Board (IRB2020-035). Written informed consent for participation was not required for this study in accordance with the national legislation and the institutional requirements.

## Author contributions

BW, BH, CAK, LS, and RM: conceptualization. BW and CAK: methodology and formal analysis. BW: software and writing—original draft preparation. CAK, BH, LS, and RM: supervision and writing—review and editing. All authors have read and agreed to the published version of the manuscript.
