# Supplementary material for: The association between socioeconomic distress communities index and amputation among patients with peripheral artery disease
Source: Front Cardiovasc Med. 2022 Nov 3;9:1021692. doi: 10.3389/fcvm.2022.1021692 (PMC9668855; doi:10.3389/fcvm.2022.1021692)
Supplement: Supplementary file 1 [file Table_1.DOCX]

Supplementary Material

# Supplementary Data

ICD-9-CM and ICD-10-CM Diagnosis Codes for Peripheral Artery Disease

| **Condition** | **ICD-9 Codes** | **ICD-10 Codes** |
| --- | --- | --- |
| Critical Limb Ischemia | 440.22, 440.23, 440.24, 707.10,  707.11, 707.12, 707.13, 707.14,  707.15, 707.16, 707.17, 707.18,  707.19, 785.4 | I70.229, I70.25, I70.269, L97.909, L97.109, L97.209, L97.309, L97.409, L97.509, L97.809, I96 |
| Peripheral Artery Disease | 440.2, 440.20, 440.21, 440.22, 440.23, 440.24, 440.29, 440.3, 440.0, 440.30, 440.31, 440.32, 440.9, 249.7, 249.71, 250.7, 250.71, 250.72, 250.73, 443.1,  443.81, 443.9, 444.22, 444.81, 785.4 | I70.209, I70.219, I70.229, I70.25, I70.269, I70.299, I70.0, I70.399, I70.499, I70.599, I70.90, I70.91, E08.51, E09.51, E13.59, E08.51, E08.65, E09.51, E11.51, E10.51, E11.51, E11.65, E10.51, E10.65, I73.1, I79.8, I73.9, I74.5, I74.3, I96 |

ICD-9-CM and ICD-10-CM Diagnosis Codes for Comorbidities

| **Condition** | **ICD-9 Codes** | **ICD-10 Codes** |
| --- | --- | --- |
| Diabetes | 249, 250, 250.0, 250.1, 250.2, 250.3, 250.4, 250.5, 250.6, 250.7, 250.8, 250.9 | E100, E101, E106, E108, E109, E110, E111, E116, E118, E119, E120, E121, E126, E128, E129, E130, E131, E136, E138, E139, E140, E141, E146, E148, E149, E102, E103, E104, E105, E107, E112, E113, E114, E115, E117, E122, E123, E124, E125, E127, E132, E133, E134, E135, E137, E142, E143, E144, E145, E147 |
| Chronic Obstructive Pulmonary Disease | 490, 491.0, 491.1, 491.2, 491.21, 491.22, 491.8, 491.9, 492, 492.8, 494, 494.0, 494.1, 496, 493.00, 493.01, 493.02, 493.10, 493.11, 493.12, 493.20, 493.21, 493.22, 493.81, 493.82, 494.90, 493.90, 493.92 | J40, J41, J42, J43, J44, J45, J47 |
| Hypercholesterolemia | 272 | E78, E881, E7521, E7522, E75249, E770, E771, E8889 |
| Renal Failure | 403.01, 403.11, 403.91, 404.02, 404.03, 404.12, 404.13, 404.92, 404.93, 585.5, 585.6, 586, 593.81, V45.11, V56 | I120, I1311, I132, N185, N186, N19, N280, Z992, Z4931, Z4901, Z4902, Z4932 |
| Chronic Kidney Disease | 249.4, 250.4, 403, 403.00, 403.10, 403.90, 404, 404.00, 404.01, 404.10, 404.11, 404.90,  404.91, 581, 581.8, 582, 583, 585.1-585.4, 585.9 | E0821, E0921, E0865, E1122, E1129, E1029, E1022, E1121, E1165, E1021, E1065, I129, I120, I130, I1311, I132, I1310, N044, N022, N043, N040, N08, N048, N049, N032, N033, N034, N038, N08, N039, N059, N052, N055, N171, N172, N058, I129, I1310, I130, N181, N182, N183, N184, N189 |
| Congestive Heart Failure | 398.91, 402.01, 402.11, 402.91, 404.01, 404.03 404.11, 404.91, 404.13, 404.93, 425.4, 425.5, 425.7, 425.8, 425.9, 428 | I43,I50,I099,I110,I130,I132,I255,I420,I425,I426, I427,I428,I429,P290 |
| Hypertension | 401,402,403,404,405 | I10, I11, I12, I13, I14, I15, I16 |
| Coronary Artery Disease | 410, 411, 412, 14, 414.0, 429.0, 29.1, 429.2, 429.3, 29.4, 429.5, 429.6, 29.7, 429.71,429.79, 429.8, 429.81, 429.82, 429.89, 429.9, v45.81, V45.82 | I2109, I2119, I2111, I2129, I214, I213, I219, I21A1, I21A9, I241, I200, I240, I248, I252, I2510, I25810, I25811, I25812, I253, I2541, I2542, I2582, I2584, I255, I2589, I259, I514, I515, I517, I970, I97110, I97130, I97190, I511, I512, I510, I230, I5189, I513, I519, Z951, Z955, Z9861 |
| Chronic Limb Threatening Ischemia | 440.22, 440.23, 440.24, 707.10,  707.11, 707.12, 707.13, 707.14,  707.15, 707.16, 707.17, 707.18,  707.19, 785.4 | I70.229, I70.25, I70.269, L97.909, L97.109, L97.209, L97.309, L97.409, L97.509, L97.809, I96 |

ICD-9-CM and ICD-10-CM Diagnosis Codes for Charlson Comorbidity Index*

| **Comorbidities** | **Points** | **Enhanced ICD-9 Codes** | **ICD-10 Codes** |
| --- | --- | --- | --- |
| Myocardial infarction | 1 | 410.x, 412.x | I21.x, I22.x, I25.2 |
| Congestive heart failure | 1 | 398.91, 402.01, 402.11, 402.91,  404.01, 404.03, 404.11, 404.13,  404.91, 404.93, 425.4-425.9, 428.x | I09.9, I11.0, I13.0, I13.2, I25.5, I42.0, I42.5-I42.9, I43.x, I50.x, P29.0 |
| Peripheral vascular disease | 1 | 093.0, 437.3, 440.x, 441.x,  443.1-443.9, 447.1, 557.1,  557.9, V43.4 | I70.x, I71.x, I73.1, I73.8, I73.9, I77.1, I79.0, I79.2, K55.1, K55.8, K55.9, Z95.8, Z95.9 |
| Cerebrovascular disease | 1 | 362.34, 430.x-438.x | G45.x, G46.x, H34.0, I60.x-I69.x |
| Dementia | 1 | 290.x, 294.1, 331.2 | F00.x-F03.x, F05.1, G30.x, G31.1 |
| Chronic Pulmonary Disease | 1 | 416.8, 416.9, 490.x-505.x, 506.4, 508.1, 508.8 | I27.8, I27.9, J40.x-J47.x, 60.x-J67.x, J68.4, J70.1, J70.3 |
| Rhematic Disease | 1 | 446.5, 710.0-710.4, 714.0-714.2, 714.8, 725.x | M05.x, M06.x, M31.5, M32.x-M34.x, M35.1, M35.3, M36.0 |
| Peptic Ulcer Disease | 1 | 531.x-534.x | K25.x-K28.x |
| Mild Liver Disease | 1 | 070.22, 070.23, 070.32, 070.33,  070.44, 070.54, 070.6, 070.9,  570.x, 571.x, 573.3, 573.4, 573.8, 573.9, V42.7 | B18.x, K70.0-K70.3, K70.9,  K71.3-K71.5, K71.7, K73.x, K74.x, K76.0, K76.2-K76.4, K76.8, K76.9, Z94.4 |
| Diabetes without complication | 1 | 250.0-250.3, 250.8, 250.9 | E10.0, E10.l, E10.6, E10.8, E10.9, E11.0, E11.1, E11.6, E11.8, E11.9, E12.0, E12.1, E12.6, E12.8, E12.9, E13.0, E13.1, E13.6, E13.8, E13.9,  E14.0, E14.1, E14.6, E14.8, E14.9 |
| Diabetes with chronic complication | 2 | 250.4-250.7 | E10.2-E10.5, E10.7, E11.2-E11.5, E11.7, E12.2-E12.5, E12.7, E13.2-E13.5, E13.7, E14.2-E14.5, E14.7 |
| Hemiplegia or paraplegia | 2 | 334.1, 342.x, 343.x, 344.0-344.6, 344.9 | G04.1, G11.4, G80.1, G80.2, G81.x, G82.x, G83.0-G83.4, G83.9 |
| Renal disease | 2 | 403.01, 403.11, 403.91, 404.02,  404.03, 404.12, 404.13, 404.92,  404.93, 582.x, 583.0-583.7, 585.x, 586.x, 588.0, V42.0, V45.1, V56.x | I12.0, I13.1, N03.2-N03.7,  N05.2-N05.7, N18.x, N19.x, N25.0, Z49.0-Z49.2, Z94.0, Z99.2 |
| Any malignancy, including lymphoma and leukemia, except  malignant neoplasm of skin | 2 | 140.x-172.x, 174.x-195.8,  200.x-208.x, 238.6 | C00.x-C26.x, C30.x-C34.x,  C37.x-C41.x, C43.x, C45.x-C58.x, C60.x-C76.x, C81.x-C85.x, C88.x, C90.x-C97.x |
| Moderate or severe liver disease | 3 | 456.0-456.2, 572.2-572.8 | I85.0, I85.9, I86.4, I98.2, 70.4, K71.1, K72.1, K72.9, K76.5, K76.6, K76.7 |
| Metastatic solid tumor | 6 | 196.x-199.x | C77.x-C80.x |
| AIDS/HIV | 6 | 042.x-044.x | B20.x-B22.x, B24.x |

Components of the Distressed Communities Index

| **Component** | **Definition** | **Source** |
| --- | --- | --- |
| No High School Diploma | Percent of the population 25+ without a  high school diploma | American Communities Survey 5-year Estimates, 2014 - 2018 |
| Poverty Rate | Percent of the population living under  the poverty line | American Communities Survey 5-year Estimates, 2014 - 2018 |
| Adults Not Working | Percent of the adults (25-64) not currently employed | American Communities Survey 5-year Estimates, 2014 - 2018 |
| Median Income | Ratio of a geography’s median income to that of its state | American Communities Survey 5-year Estimates,  2014 - 2018 |
| Housing Vacancy Rate | Percent of habitable housing that is  unoccupied, excluding properties  that are for seasonal, recreational, or  occasional use | American Communities Survey 5-year Estimates, 2014 - 2018 |
| Change in Establishments | Percent change in the number of  business establishments | Census Bureau County and ZIP Code Business Patterns,  2014 - 2018 |
| Change in Employment | Percent change in the number of jobs | Census Bureau County and ZIP Code Business Patterns,  2014 - 2018 |

ICD-9-CM and ICD-10-CM Diagnosis Codes for Amputations

| Major Limb Amputations | | Minor Limb Amputations | |
| --- | --- | --- | --- |
| ICD 9 Procedure Codes | ICD 10 Procedure Codes | ICD 9 Procedure Codes | ICD 10 Procedure Codes |
| - Lower limb (84.10) - Amputation of ankle (84.14) - Other amputation below knee (84.15) - Disarticulation of knee (84.16) - Amputation above knee (84.17) - Disarticulation of hip (84.18) - Abdominopelvic amputation (84.19) | - Hindquarter (0Y62, 0Y63, 0Y64) - Femoral region (0Y67 or 0Y68) - Upper leg (0Y6C or 0Y6D) - Knee region (0Y6F or 0Y6G) - Lower leg (0Y6H or 0Y6J) | - Amputation of toe (84.11) - Amputation through foot (84.12) - Disarticulation of ankle (84.13) | - Detachment of foot (0Y6M or 0Y6N) - Detachment of toe (0Y6P - 0Y6Y) |

**Table 2.** Adjusted subdistribution hazard ratios of major amputation

|  | **All patients**  **(82,848)** | **Non-Hispanic White**  **(57,744)** | **Non-Hispanic Black**  **(25,104)** |
| --- | --- | --- | --- |
| Distressed Community Index |  |  |  |
| DCI < 40 | Ref | Ref | Ref |
| DCI ≥ 40 | 1.25 (1.14, 1.37) | 1.14 (1.01, 1.29) | 1.36 (1.18, 1.57) |
| Age | 1.01 (1.01, 1.01) | 0.99 (0.99, 1.00) | 1.02 (1.01, 1.02) |
| Sex |  |  |  |
| Male | Ref | Ref | Ref |
| Female | 0.63 (0.59, 0.68) | 0.67 (0.59, 0.76) | 0.60 (0.54, 0.66) |
| Race/Ethnicity |  |  |  |
| Non-Hispanic White | Ref |  |  |
| Non-Hispanic Black | 2.96 (2.73, 3.21) |  |  |
| Primary Insurer |  |  |  |
| Private | Ref | Ref | Ref |
| Medicare | 1.35 (1.18, 1.54) | 1.39 (1.14, 1.71) | 1.39 (1.15, 1.67) |
| Medicaid | 1.77 (1.49, 2.10) | 2.10 (1.6, 2.77) | 1.76 (1.41, 2.20) |
| No Insurance | 1.29 (1.06, 1.59) | 1.50 (1.11, 2.01) | 1.20 (0.91, 1.58) |
| Other | 1.34 (1.11, 1.63) | 1.42 (1.08, 1.88) | 1.31 (1.01, 1.70) |
| Diabetes | 1.00 (0.92, 1.09) | 1.07 (0.94, 1.21) | 0.94 (0.84, 1.04) |
| Renal Failure | 1.40 (1.27, 1.55) | 1.22 (1.00, 1.48) | 1.51 (1.34, 1.70) |
| COPD | 0.69 (0.62, 0.77) | 0.69 (0.59, 0.80) | 0.69 (0.60, 0.80) |
| CHF | 0.85 (0.77, 0.93) | 0.91 (0.78, 1.06) | 0.81 (0.73, 0.92) |
| CAD | 0.95 (0.87, 1.03) | 0.99 (0.77, 1.01) | 1.02 (0.91, 1.14) |
| CLTI | 3.89 (3.58, 4.22) | 4.36 (3.82, 4.96) | 3.51 (3.16, 3.89) |
| CCI | 1.13 (1.11, 1.15) | 1.15 (1.12, 1.18) | 1.11 (1.09, 1.14) |

Subdistribution Hazard Ratio (95% Confidence Interval); COPD=chronic obstructive pulmonary disease; CHF=congestive heart failure; CAD=coronary artery disease; CLTI=chronic limb threatening ischemia; CCI=Charlson comorbidity index
